# High-throughput, pan-leukocyte biomarkers for the detection of inflammation in human breastmilk and stool

**DOI:** 10.3389/freae.2025.1535838

**Published:** 2025-02-28

**Authors:** M. J. Dunnet, I. M. Morison, D. M. Bond, T. A. Hore

**Affiliations:** ^1^ Department of Anatomy, University of Otago, Dunedin, New Zealand; ^2^ Department of Biochemistry, University of Otago, Dunedin, New Zealand; ^3^ Department of Pathology, University of Otago, Dunedin, New Zealand

**Keywords:** DNA methylation, biomarker, leukocytes, inflammation, mastitis, inflammatory bowel disease

## Abstract

**Background:**

DNA methylation can be used to track cellular identity. We have previously developed a high-throughput, cost-effective DNA methylation pipeline containing two loci, *HOXA3* and *MAP4K1*, that can quantify leukocyte proportion amongst a range of background tissues. Here, we apply this pipeline to two clinically relevant tissue samples: breastmilk and stool.

**Results:**

We report that our leukocyte methylation assay can quantify the proportion of leukocytes in breastmilk, and find leukocyte levels fluctuate dramatically in concert with infection severity. We benchmarked our leukocyte methylation pipeline in stool samples against the commonly used faecal calprotectin assay. Our results show a high concordance between the two methods indicating the viability of our DNA methylation biomarkers in the context of intestinal inflammation.

**Conclusion:**

The data presented here emphasise the clinical applicability of our high-throughput DNA methylation assay in the context of mastitis and intestinal inflammation.

## Introduction

DNA methylation is the covalent bonding of a methyl group to the 5′ carbon of a cytosine nucleotide. DNA methylation is dynamically added and removed throughput development; indeed, approximately 20% of all human autosomal CpG sites are differentially methylated or unmethylated based upon cell type and stage of development (Ziller et al., 2013). As such, cell-specific DNA methylation patterns can be used to identify the cell-of-origin for a particular DNA molecule. In cases where there is no genetic difference between healthy and diseased tissue or where information on cellular origin is required, DNA methylation assays will likely be useful for detection, diagnosis, and management decisions. For example, cell-free DNA (cfDNA), which can originate from apoptotic or necrotic cells, can enter the circulatory system and be collected easily and non-invasively for diagnostic purposes. Analysis of the methylation pattern of cfDNA in healthy individuals shows its cellular origins: 55% from leukocytes, 30% from erythrocyte progenitors, 10% from vascular endothelial cells, and 1% from hepatocytes (Moss et al., 2018; Lam et al., 2020). In addition, in cases of trauma, autoimmunity, ischaemia, infection, or cancer, previously undetectable cfDNA molecules from affected organs can be measured (Lehmann-Werman et al., 2016; Cisneros-Villanueva et al., 2022; Zemmour et al., 2018; Lehmann-Werman et al., 2018; Cheng et al., 2021).

Local inflammation is difficult to detect with cfDNA because of the large amount of blood-cell-derived DNA already present in plasma. Several deconvolution algorithms based on Illumina 450K methylation array and EPIC array systems have been utilised for this analysis; for example, EPIDish (Teschendorff et al., 2017), can determine the proportion of leukocyte subpopulations from a given sample. However, these systems require thousands of CpG sites, are relatively expensive, and are low throughput. We have previously described a high throughput, cost-effective, pipeline that uses the locus-specific methylation to detect immune cells from a mixed sample (Dunnet et al., 2022). This involves bisulfite amplicon sequencing of *HOXA3* and *MAP4K1* (Dunnet et al., 2022). To expand on this work here, we examined the ability of this pipeline to infer the level of inflammation from clinically relevant samples, specifically, breastmilk and stool. These samples were chosen because of their relevance to common inflammatory conditions: mastitis and inflammatory bowel disease.

Mastitis is defined as the inflammation of the breast tissue, which typically, but not exclusively, occurs during lactation (Boakes et al., 2018). The fraction of leukocytes markedly increases from less than 5% in healthy mothers to up to 90% during mastitis (Hassiotou et al., 2013a). Flow cytometry approaches to measure the leukocyte fraction in breastmilk have already been proposed as a diagnostic tool to assess the health status of the mother/infant dyad (Hassiotou et al., 2013a). However, cellular composition in breastmilk is highly dynamic and sampling may be required more frequently than flow cytometry can conveniently provide.

Intestinal inflammation can be caused by a large number of factors, including cancer (von Roon et al., 2007), inflammatory bowel disease (Laserna-Mendieta and Lucendo, 2019), and coeliac disease (Ertekin et al., 2010). Detection of intestinal inflammation is most commonly performed with the faecal calprotectin assay (FCA), which measures the concentration of calprotectin, an antimicrobial complex highly abundant in the granules of neutrophils and, to a lesser extent in monocytes and macrophages (Odink et al., 1987; Røseth PNSMKF, 1999). The amount of calprotectin is proportional to the severity of an immune response, making it a useful non-invasive marker for intestinal inflammation (Røseth et al., 1992). While an extremely valuable tool, the FCA is limited in several ways. Firstly, the FCA kits are proprietary, and the inter-kit variability is large (Whitehead et al., 2013; Kittanakom et al., 2017; Labaere et al., 2014). Second, the calprotectin complex degrades in stool after 48–72 h at room temperature and requires refrigeration to remain stable (Labaera et al., 2014; Lasson et al., 2015; Oyaert et al., 2017). Finally, the age of the patient, medications they are taking, and some pathologies (in particular, pancreatic insufficiency) can result in variation in faecal calprotectin levels (Laserna-Mendieta and Lucendo, 2019; Degraeuwe et al., 2015; Henderson et al., 2014; Padoan et al., 2018; Lundgren et al., 2019; Ellemunter et al., 2017).

The high-throughput DNA methylation biomarker pipeline we have previously described (Dunnet et al., 2022) has the potential to address limitations in the current diagnostic assays. In particular, the ability to sample repeatedly, to avoid proprietary kits, and to capitalize on the stability of DNA methylation have the potential to improve the diagnostic capabilities for mastitis and intestinal inflammation.

In this study, we sampled numerous breastmilk samples from the same individual over the course of 1 month. We show the proportion of leukocytes varied markedly, even within a 24-hour period. The two biomarker loci, *HOXA3* and *MAP4K1*, were strongly correlated in milk samples, and the total level of estimated leukocytes was consistent with instances of mastitis. Furthermore, we examined the leukocyte proportion in stool samples with associated faecal calprotectin scores. We observed that the proportion of leukocyte-derived reads was extremely high (>70%) in all samples with even slightly elevated faecal calprotectin, suggesting that leukocyte DNA vastly exceeds epithelial cell DNA in stool with even minor inflammation. Our high-throughput, cost-effective DNA methylation assay has clinical relevance in the context of mastitis and intestinal inflammation.

## Methods

### Human breastmilk sample collection and cell isolation

Human breastmilk was obtained from a single donor with informed consent per the New Zealand Human Tissue Act 2008. Over 1 month, from the 22nd of November 2018 to the 21st of December 2018, 1 mL of excess pump-expressed milk was collected by the donor where possible. Pumping occurred either exclusively from one breast or was a mixture of the two breasts; while breast of origin was often recorded by the donor, in many cases it was not. The start of the collection date was approximately 5 weeks postpartum and seventy-five milk samples were collected in total. Milk was stored at −20°C, or colder, until use.

Cell isolation was performed by centrifugation. First, milk samples were centrifuged at 500 x g for 15 min to pellet the cells. Next, milk fats and liquid were removed, and the pellet was resuspended in 500 μL of 0.01 M PBS. Centrifugation and washing were repeated an additional two times. Finally, the cells were pelleted at 500x g for 15 min before resuspension in a lysis buffer consisting of 8 μL of 0.01 M PBS, 8 μL of the Zymo 2x M-Digestion Buffer, and 1 μL of 20 mg/mL Proteinase K (Zymo EZ-96 DNA Methylation-Direct™ MagPrep Kit) and incubated at 50°C for 20 min. Cell lysates were directly added to the bisulphite conversion protocol (see below) with no additional handling.

### Saliva cell isolation and DNA extraction

Saliva samples were collected as previously described (Dunnet et al., 2022; Theda et al., 2018). Briefly, in the 30 min prior to collection only water was consumed. Five mL of saliva was collected via passive drool and transferred to a 15 mL plastic centrifuge tube. The saliva was centrifuged at 400 g and supernatant removed to isolate the cells. The cell pellets were resuspended and washed with 0.01 M PBS three times. DNA was extracted using the BOMB.bio protocol 6.3: extraction of TNA from mammalian tissues (Oberacker et al., 2019) (bomb.bio/protocols/). DNA quality was assessed by gel electrophoresis and concentration by the HS dsDNA Qubit assay (Thermo Scientific).

### K562 cell culture and DNA extraction

K562 cells were cultured as previously described (Pencovich et al., 2011). The cells were lysed with 1 mL of GITC lysis buffer (4 M GITC, 2% w/v SDS, 50 mM Tris-HCl pH 8.0, 0.1% v/v antifoam 204 (Sigma-Aldrich), and 20 mM EDTA). DNA extraction was performed with the BOMB.Bio protocol 6.1: TNA extraction of mammalian cells with GITC (Oberacker et al., 2019) (bomb.bio/protocols/). DNA quality was assessed by gel electrophoresis and concentration by the HSdsDNA Qubit assay.

### Leukocyte isolation and DNA extraction

Leukocytes were isolated from saliva (see above) by sequential cellular filtration as previously described (Dunnet et al., 2022). Briefly, cellular isolates from saliva were first filtered through a 40-μm, then a 20-μm mesh filter to exclude buccal cells. The purity of the isolated leukocytes was assessed by microscopy. We counted a minimum of 100 cells across two fields per slide. We observed a purity of >99% for each sample.

### Stool sample preparation and DNA extraction

The use of human stool samples was approved by the University of Otago Human Ethics Committee (Health) (approval number H21/138). Stool samples were obtained from Southern Community Laboratories (SCL), Dunedin. SCL had previously processed the stool samples for use in a FCA; specifically, they were homogenised and diluted in a proprietary extraction buffer. After the faecal calprotectin samples were received, they were heat-inactivated at 75 °C before DNA extraction with the Zymo Research™ Quick-DNA Fecal/Soil Microbe Miniprep Kit. DNA extractions were performed starting at step four of the protocol (steps 1-3 are for homogenisation). Furthermore, to improve overall DNA yield per sample, the volume of homogenised stool sample and genomic lysis buffer was increased four-fold from 400 μL to 1,200 μL to 1,600 μL and 4,800 μL, respectively. The entirety of each sample was run through the extraction column over multiple spin cycles. The remainder of the DNA extraction was performed as above. Other than these modifications, extraction was carried out as per the manufacturer’s instructions. Total DNA concentration was measured with the HS dsDNA Qubit assay, and DNA quality was assessed by gel electrophoresis.

### Bisulfite conversion of DNA

Bisulphite conversion for all samples was performed with the Zymo EZ-DNA Methylation Direct MagPrep kit per the manufacturer’s instructions. This protocol suggests various initial conversion parameters specific to the amount and quality of input DNA. Lysates with breastmilk-derived DNA were converted under standard conditions for optimal cytosine conversion: 8 min at 98°C followed by 3 h and 30 min at 64°C. Stool-derived DNA samples were converted for 8 min at 98°C followed by 3 h and 30 min at 53°C to reduce DNA degradation at the cost of less efficient conversion. Bisulfite converted DNA was quantified using the Qubit ssDNA Qubit assay (Thermo Scientific).

### Bisulfite amplicon sequencing

A dual-index, two-step PCR protocol was used to amplify bisulphite-converted DNA with the KAPA HiFi HotStart Uracil + kit (Roche) as described here (Dunnet et al., 2022). Briefly, in the first round of PCR, the target region is amplified with primers containing an overhanging linker sequence. The second round of PCR uses primers comprised of the complementary linker sequence attached to the Illumina P5 and P7 adapters and TruSeq indexes. Each PCR reaction contained the KAPA HiFi HotStart Uracil + ReadyMix, 0.3 μM of each primer, and 200 ng of bisulphite-converted DNA topped up to 25 μL with nuclease-free water. All primer sequences are shown in Table 1.

**TABLE 1:** The primer sequences used in this study. Bold text indicates linker sequences for bisulphite amplicon sequencing. Note that this linker sequence is the reverse complement of the P5 and P7 adapters.

Primer ID	Sequence	Target strand	Target location (hg38)
Bisulfite HOXA3_long Forward	**ACA​CTC​TTT​CCC​TAC​ACG​ACG​CTC​TTC​CGA​TCT​**GGT​TTT​GTT​TGG​GTT​AGT​GGT​AT	+	chr7:27,113,959-27,114,117
Bisulfite HOXA3_long Reverse	**GTG​ACT​GGA​GTT​CAG​ACG​TGT​GCT​CTT​CCG​ATC​T**CC​AAC​AAA​AAA​ACC​CCT​TTA​TAA​A	+	chr7:27,113,959-27,114,117
Bisulfite MAP4K1_long Forward	**ACA​CTC​TTT​CCC​TAC​ACG​ACG​CTC​TTC​CGA​TCT​**TGT​TTT​TAT​ATG​GAA​GTT​ATA​TTT​ATT	-	chr19:38,596,411–38,596,696
Bisulfite MAP4K1_long Reverse	**GTG​ACT​GGA​GTT​CAG​ACG​TGT​GCT​CTT​CCG​ATC​T**AA​CAA​CTC​AAA​ACC​TAA​CCC	-	chr19:38,596,411–38,596,696
Bisulfite MAP4K1_short Forward	**ACA​CTC​TTT​CCC​TAC​ACG​ACG​CTC​TTC​CGA​TCT​**TTA​GAA​ATG​TTA​GGG​GAT​AAG​GTT​T	+	chr19:38,596,606-38,596,816
Bisulfite MAP4K1_short Reverse	**GTG​ACT​GGA​GTT​CAG​ACG​TGT​GCT​CTT​CCG​ATC​T**TT​CCT​CAA​ACT​CAA​TAC​TAC​CAC​TC	+	chr19:38,596,606-38,596,816
Illumina P5 adapter with TruSeq index	AATGATACGGCGACCACCGAGATCTACACNNNNNN**ACACTCTTTCCCTACACGACGCTCTTCCGATCT**	N/A	N/A
Illumina P7 adapter with TruSeq index	CAAGCAGAAGACGGCATACGAGATNNNNNN**GTGACTGGAGTTCAGACGTGTGCTCTTCCGATCT**	N/A	N/A

Breastmilk-derived DNA was first amplified with the *HOXA3_long* and *MAP4K1_long* primer pairs. The following cycle parameters were used: 95°C for 2 min, 23 cycles of 98°C for 20 s, 59°C for 10 s, and 72°C for 20 s. A final elongation step was performed for 5 min at 72°C. Reactions were centrifuged briefly to remove condensate on the tube walls. Next, the products were cleaned using solid-phase reverse immobilisation of carboxyl-coated magnetic beads suspended in standard PEG buffer (18% w/v polyethylene glycol 8000 (PEG), 1 M NaCl, 10 mM Tris (pH 8.0), 1 mM EDTA, 0.05% v/v Tween-20), followed by two washes in 70% ethanol. The cleaned PCR products were eluted in 11.5 μL of filter sterile Milli-Q^®^ water. The eluted DNA was combined with 12.5 μL of the KAPA HiFi HotStart Uracil + ReadyMix and 0.5 μL of each indexing primer (Illumina P5 and P7 adapters with TruSeq index combined with the complementary linker sequence). Amplification was repeated as above for an additional five cycles.

Saliva, K562, and human stool-derived DNA were amplified with the *MAP4K1_short* primer pair. The same approach as above was followed with modifications to thermocycling to improve PCR efficiency. The first amplification step was performed with the following parameters: 95°C for 2 min, 25 cycles of 98°C for 20 s, 59°C for 40 s, and 72°C for 40 s. A final elongation step was performed for 5 min at 72 °C. The PCR product solid-phase reverse immobilisation clean-up was performed similarly as above. The second amplification step used the following parameters: 95°C for 2 min, 5 cycles of 98°C for 20 s, 59°C for 40 s, and 72°C for 40 s, followed a final 5 min elongation step at 72°C.

Prior to sequencing, all amplicons were cleaned using solid-phase reverse immobilisation of carboxyl-coated magnetic beads suspended in standard PEG buffer (as above). Sequencing was performed on the Illumina iSeq100 as per the manufactures instructions.

### Bioinformatic and statistical analyses

Raw read adapter and quality trimming were performed with Cutadapt and TrimGalore (v.0.6.7) (Martin, 2011). Reads were mapped to a custom “genome” consisting of only the amplicon sequence using Bismark (v0.14.3) (Krueger and Andrews, 2011). The sequences used for mapping were obtained from the UCSC genome browser (*HOXA3_long*: chr7:27,113,957–27,114,300 (hg38); *MAP4K1_long:* chr19:38,596,411–38,596,696 (hg38); *MAP4K1_short*: chr19:38,596,606-38,596,816 (hg38)). Heatmaps, linear regression, bionomical regression, cell-of-origin read classification, and ROC curve generation were performed with custom R scripts and the pROC package. The cut-off for a read to be classified as leukocyte derived was ≥6 methylated CpG sites for the *HOXA3_long* amplicon, ≥3 for the *MAP4K1_long* amplicon, and ≥4 for the *MAP4K1_short* amplicon. All statistical analyses were performed in R.

## Results

### DNA methylation at the *HOXA3* and *MAP4K1* loci can determine the proportion of blood-derived cells in human breastmilk.

Human breastmilk contains three principal cell populations (Ziller et al., 2013): blood-derived cells, comprised of both mature leukocytes and hematopoietic stem cells (Moss et al., 2018), breast-derived cells, comprised of lactocytes, myoepithelial cells, and progenitor cells; and (Lam et al., 2020) probiotic bacteria (Witkowska-Zimny and Kaminska-El-Hassan, 2017). In a healthy mother/child dyad, the proportion of leukocytes is high in the colostrum (between 13% and 70%), defined as the first milk until approximately 4 days postpartum, but rapidly decreases after that to less than 1% of the total cells in breastmilk (Hassiotou et al., 2013a; Witkowska-Zimny and Kaminska-El-Hassan, 2017). In contrast, when either the mother or child is ill, leukocyte proportions increase; during mastitis, leukocytes can constitute upwards of 90% of total breastmilk cells (Hassiotou et al., 2013a). Therefore we first aimed to determine if the previously described *HOXA3* and *MAP4K1* DNA methylation biomarkers (Dunnet et al., 2022) can accurately deconvolute blood-derived cells from breast-derived cells in human breastmilk. Seventy-five breastmilk samples from a single individual were gathered over the course of 1 month with various levels of meta-data (Figure 1A). Samples were recorded as originating from either the left or right breast during the first week of collection; samples after week one were either pooled together from both breasts or had no associated information. We examined DNA methylation at the *HOXA3* and *MAP4K1* loci and applied a previously described leukocyte estimation pipeline for each sample (Dunnet et al., 2022). Reads were either highly methylated or highly unmethylated with considerable concordance between both loci (*R* ^2^ = 0.95, Figures 1B, C), suggesting both biomarkers function similarly in breastmilk.

**FIGURE 1:**
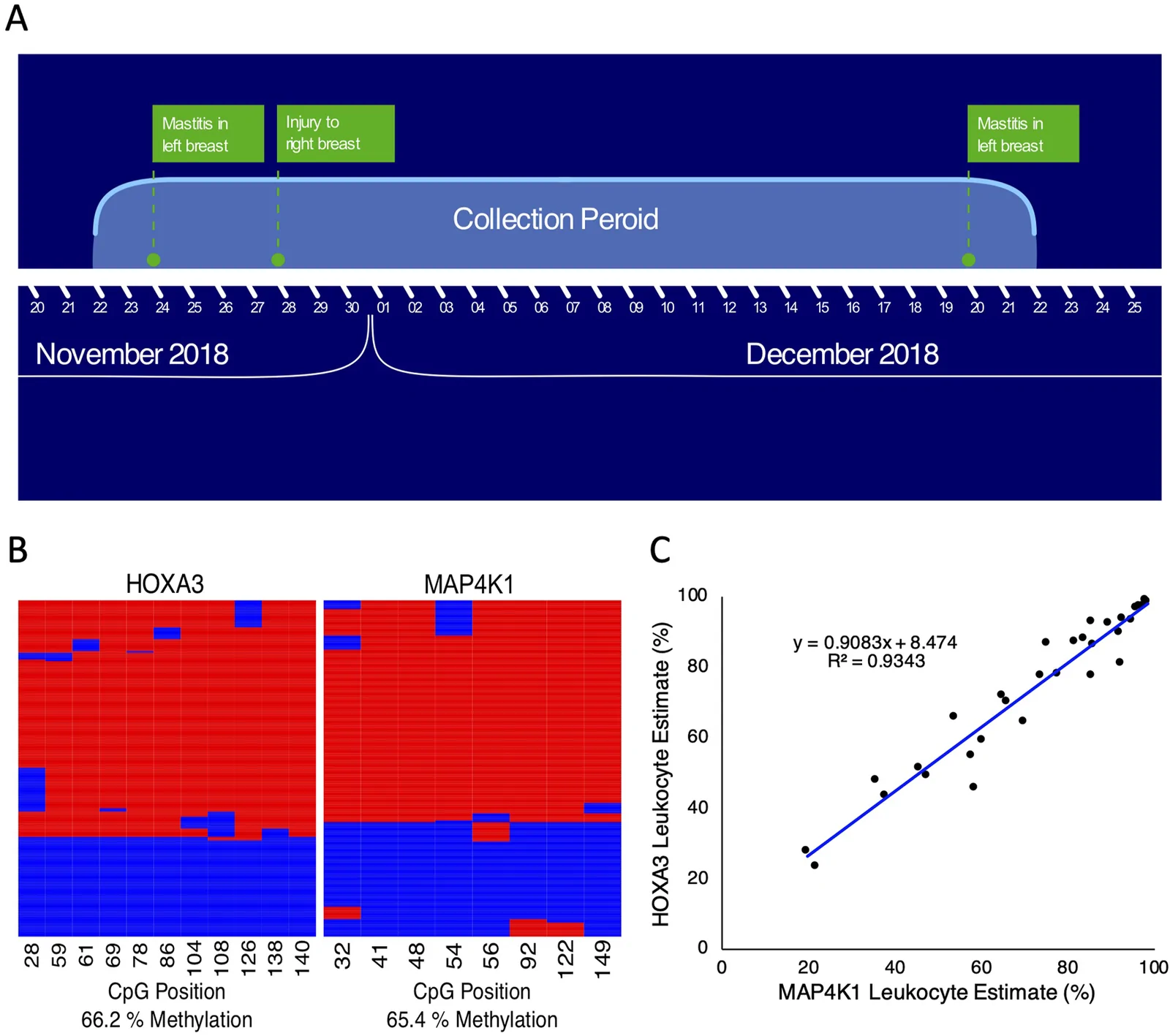
*HOXA3* and *MAP4K1* leukocyte estimates correlate in human milk samples. **(A)** Overview of the milk collection period **(B)** Example of bisulphite amplicon heatmaps for *HOXA3* (left) and *MAP4K1* (right) from human milk samples. Sequencing reads run from left to right across the rows, and columns represent individual CpG positions. Red indicates a methylated CpG, and blue indicates an unmethylated CpG. **(C)** Scatterplot of leukocyte estimates for *MAP4K1* (X-axis) vs. *HOXA3* (Y-axis).

Next, we examined the leukocyte proportions from samples with available meta-data on the breast of origin (Figure 2). During the time these samples were taken, there was mastitis in the left breast and an injury to the right breast resulting from breastfeeding. We observed a high proportion of leukocytes in the left-breast-associated milk throughout the week and elevated leukocyte proportions in right-breast-associated milk occurring at the peak of mastitis in the left breast and during the injury to the right breast (Figure 2A). Outside of these time points, the number of leukocytes in the right breast decreased to approximately 60% of the total cell population. These results suggest that during mastitis, the proportion of leukocytes is elevated primarily in the infected breast; however, a severe infection can produce elevated leukocyte proportions in both breasts regardless of where the infection is located.

**FIGURE 2:**
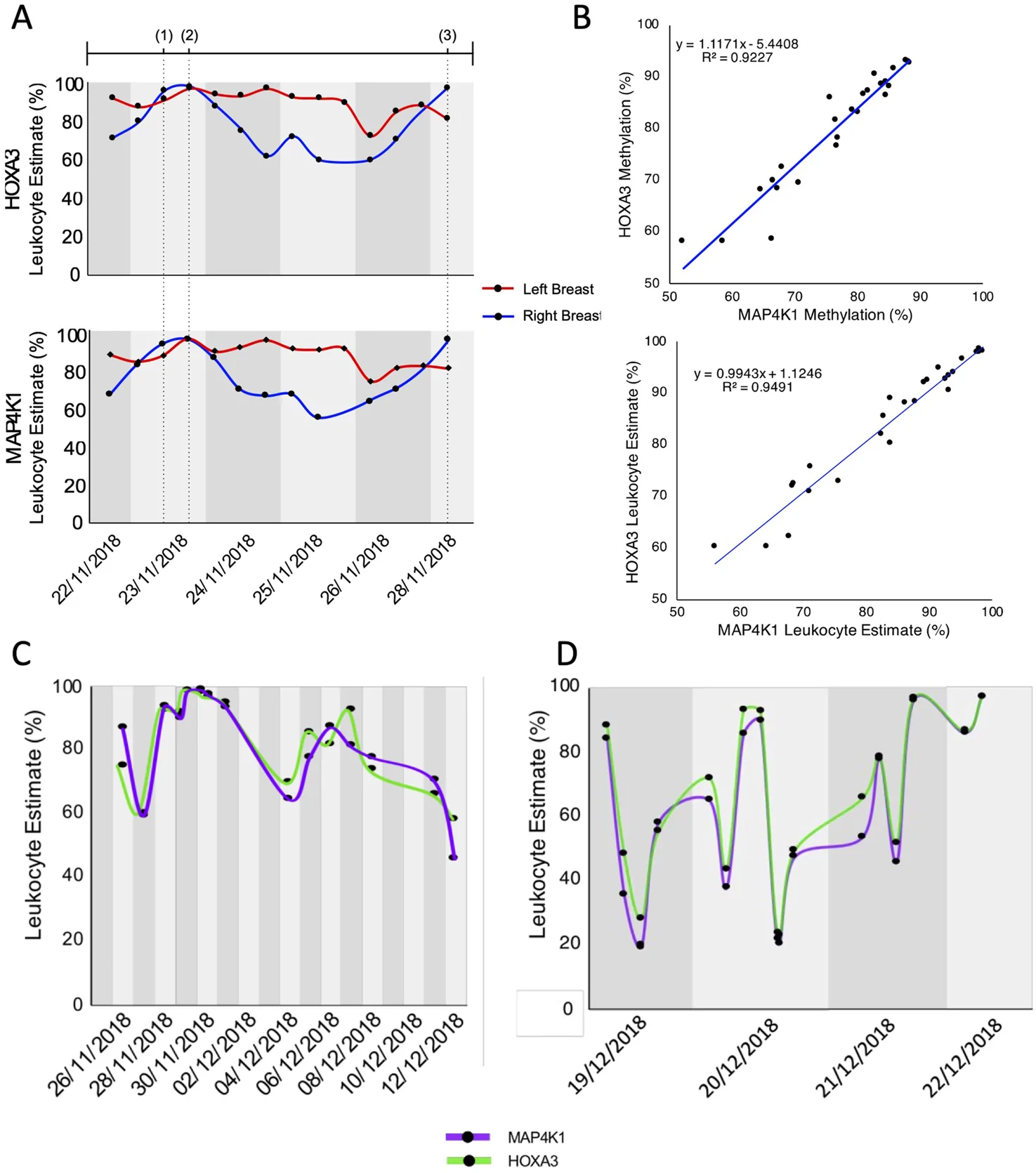
Tracking leukocytes in breastmilk over time **(A)** The proportion of leukocytes in milk from either the left (red line) or right (blue line) breast over 1 week as measured by *HOXA3* (top) and *MAP4K1* (bottom). Dotted vertical lines indicate the timepoint of events (Ziller et al., 2013): onset of flu-like symptoms as a result of left breast mastitis (Moss et al., 2018), peak of left-breast mastitis, including fever (Lam et al., 2020), injury to the right breast as a result of breastfeeding. **(B)** Scatterplot of *HOXA3* vs. *MAP4K1* methylation (top) and leukocyte estimate (bottom) for each sample with breast-of-origin meta-data. **(C)** Proportion of leukocytes measured by *HOXA3* and *MAP4K1* from milk known to be pooled from both breasts. **(D)** Proportion of leukocytes measured by *HOXA3* and *MAP4K1* from milk where breast of origin (or its mixing) was not recorded.

Throughout the month-long period of sample collection, we observed a high proportion of leukocytes. Indeed, the mean leukocyte estimation across the collection period was 72.3% for *HOXA3* (median 76.6%) and 70.9% for *MAP4K1* (median = 73.8%); the sample with the lowest leukocyte proportion was estimated at 23.5% by *HOXA3* and 21.6% by *MAP4K1*. Nevertheless, this was not unexpected because of continued mastitis in the left breast and damage to the right breast. Interestingly, leukocyte estimates did not shift dramatically on a day-to-day basis when either the breast of origin was known or the samples were comprised of milk from both breasts (Figures 2A, C); however, we observed striking changes between milk samples collected on the same day when breast-of-origin meta-data was not available (maximum difference of 69%, Figure 2D, see methods). Therefore, we hypothesise that these rapid changes in cellular composition are partly the result of milk samples obtained from different breasts. Indeed, the cellular composition of breast milk is dynamic and changes based on the period of lactation, infection status, and infant feeding habits (Hassiotou et al., 2013a; Witkowska-Zimny and Kaminska-El-Hassan, 2017; Riskin et al., 2012). It is, therefore, unsurprising that each breast’s cellular composition can differ depending on circumstance, especially in cases of infection.

### Validation of a *MAP4K1* bisulfite amplicon for application with stool-derived human DNA

Total genomic DNA extracted from stool samples contains an amalgamation of bacterial-, fungal-, viral-, and host-derived DNA. In healthy individuals, the human component constitutes less than 1% of the total DNA (Vincent et al., 2015). In contrast, chronic intestinal inflammation damages the surrounding tissues and subsequently results in cell death (Anderton et al., 2020), leading to the hypothesis that more host-derived DNA will be present in the stool. Indeed, it has been shown that both mitochondrial DNA and genomic DNA amounts increase based on the severity of inflammatory bowel disease (IBD)-related intestinal inflammation (Casellas et al., 2004; Vrablicova et al., 2020). We hypothesized that under inflammatory conditions, DNA from neutrophils that have migrated into the intestinal lumen, could be detected with our DNA methylation assay. We obtained 48 faecal samples that had previously undergone FCA testing for our analysis. We initially examined a subset of these samples with the *HOXA3* and *MAP4K1* (Dunnet et al., 2022) amplicons described above; however, we were unable to obtain sufficient amplicon copies for sequencing within 30 PCR cycles. Over-amplification with more than 30 cycles can lead to clonal amplification of only a few DNA molecules, leading to a misinterpretation of DNA methylation patterns. As a result, shorter amplicons were designed to improve PCR yield (Table 1; Bisulfite *MAP4K1_short*). We sequenced leukocyte and K562 DNA (the latter is unmethylated in the *HOXA3* and *MAP4K1* loci of interest (Dunnet et al., 2022)) to ensure the DNA methylation patterns are consistent between the short and long amplicons. As expected, we observed that leukocytes were highly methylated (84.9%), while K562 DNA was virtually devoid of DNA methylation (4.6%) (Figure 3A). The near absence of methylation of K562 cells parallels the methylation of these loci in colonic epithelial cells (Dunnet et al., 2022). The two cell types cluster separately from one another based upon the number of methylated CpG sites per read (Figure 3B). To classify reads as either derived from mature leukocyte DNA or K562 DNA we employed a binomial logistic regression model and constructed a receiver operating characteristic (ROC) curve using the ‘pROC’ package in R. The optimal threshold for leukocyte classification was if ≥4 of seven CpG sites were methylated (sensitivity = 98.3%, specificity = 99.98%, Figure 3C). Furthermore, the area under the ROC curve was 0.998, indicating that the methylation patterns obtained from this amplicon perform exceptionally well at distinguishing blood leukocyte-derived DNA from K562 DNA (Figure 3D).

**FIGURE 3:**
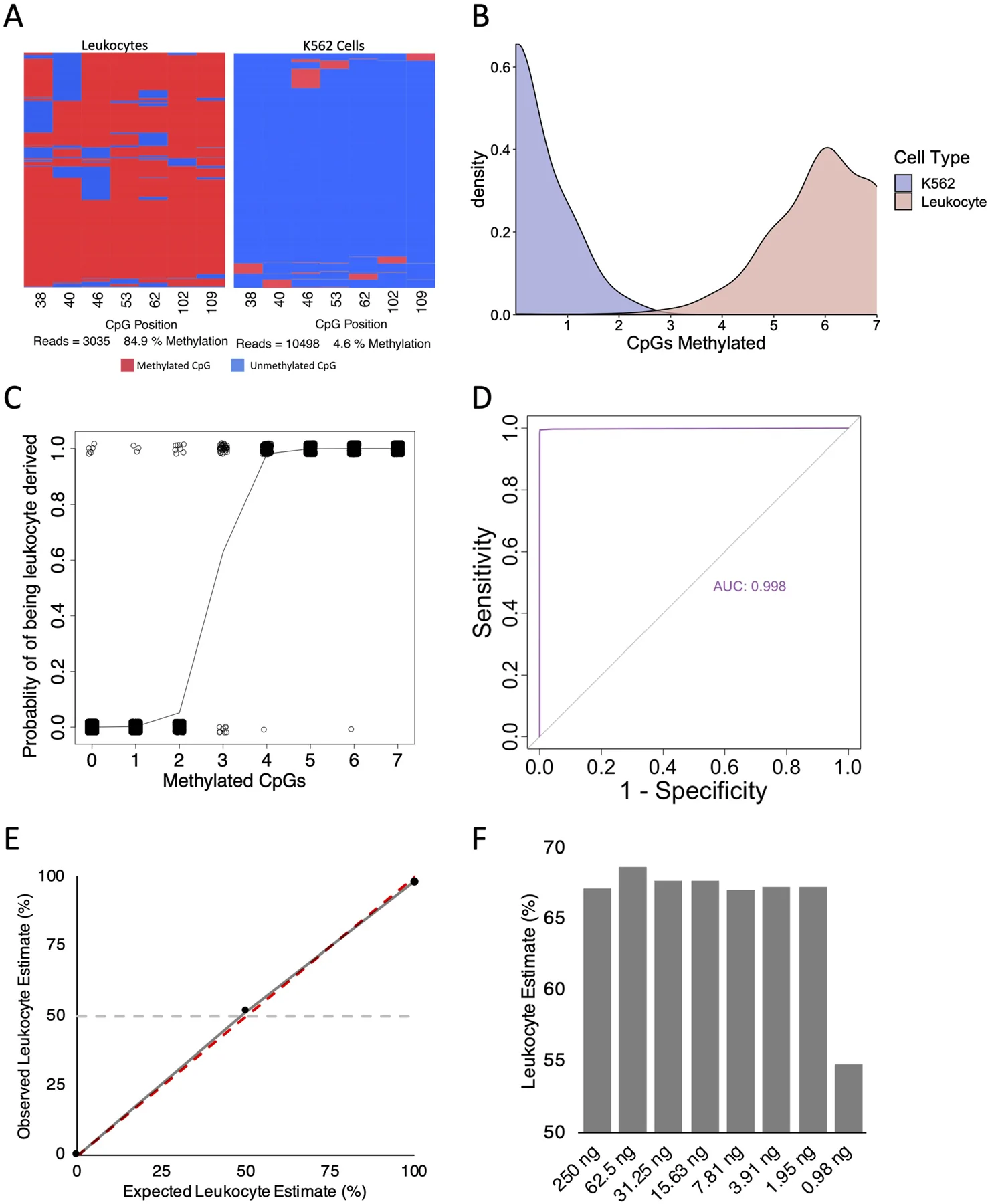
Validity of short *MAP4K1* amplicon primer sets. **(A)** Example bisulfite amplicon sequencing heatmaps from leukocyte derived DNA (left) and K562 DNA (right). **(B)** Density plot for the number of methylated CpG sites per read in K562- (blue) and Leukocyte-derived (red) DNA samples. The Y-axis represent the probability per unit on the X-axis such that the area under the curve for a specific interval is equal to the probability of the number of methylated CpG sites for that interval. Bandwidth 0.45 **(C)** Binomial regression analysis for the *MAP4K1* short amplicon using leukocyte and K562 DNA. **(D)** ROC curve for the *MAP4K1* short amplicon using leukocyte and K562 DNA. **(E)** Scatterplot of observed vs. expected read classification for leukocyte and K562 DNA mixes. The dotted red line indicates a perfect fit, the solid grey line indicates the observed trendline, and the horizontal dotted grey line indicates a 50% leukocyte estimate. **(F)** Bar chart of leukocyte estimates from two-fold, serially diluted saliva-derived DNA.

We next asked if the short *MAP4K1* PCR showed preferential amplification of methylated or unmethylated alleles and if leukocyte estimates are consistent across differing amounts of input DNA. We combined blood leukocyte derived DNA and K562 DNA at a 50:50 ratio and observed a leukocyte estimate of 51.5%. With leukocyte and K562 DNA alone we observed leukocyte estimates of 98.3% and 0.2% respectively (Figure 3E). Finally, we performed a two-fold serial dilution with saliva-derived DNA (a combination of salivary leukocytes and buccal epithelium (Theda et al., 2018)) and observed consistent levels of leukocyte estimates until 0.98 ng of DNA, where the result became discordant (Figure 3F). This suggests the assay can accurately determine the proportion of leukocyte-derived DNA with as little as 2 ng of input DNA.

### *MAP4K1* DNA methylation can identify intestinal inflammation as measured by faecal calprotectin

To determine if our bisulfite amplicon sequencing pipeline could be used to detect intestinal inflammation we applied the pipeline (with the shorter *MAP4K1* amplicon) to stool samples that had previously undergone a FCA. The cutoff for a positive faecal calprotectin test result in the laboratory we obtained samples from is 50 μg/g. However, various cut-off thresholds have been suggested in the literature to best balance sensitivity and specificity (Jha et al., 2018; Rogler et al., 2013; Ricciuto and Griffiths, 2019). The reference interval of calprotectin is 10–100 μg/g for Caucasians, depending on the kit (Bjarnason, 2017), although the interval between 50 and 100 μg/g is thought to have some diagnostic value (Ricciuto and Griffiths, 2019). In contrast, a calprotectin score of up to 200 μg/g can be considered normal for people of African-Caribbean descent (Bjarnason, 2017). Furthermore, thresholds as high as 250 μg/g have been shown to markedly increase specificity at the cost of sensitivity (Jha et al., 2018; Rogler et al., 2013). For this reason, we examined the leukocyte estimates in three discrete bands: calprotectin scores less than 50 μg/g (low), between 50 and 250 μg/g (moderate), and greater than 250 μg/g (high). Low, moderate, and high calprotectin samples had mean leukocyte estimates of 51.8% (S.D. = 28.4%), 77.9% (S.D. = 22.9%), and 94.2% (S.D. = 4.2%), respectively (Figure 4A). The percentages of leukocyte-derived DNA in stool from low calprotectin samples were extremely variable, ranging from 3.4% to 88.7%. In contrast, leukocyte estimates in moderate and high samples were extremely similar and all above 70% (with the exception of one moderate sample with a calprotectin level of 52 μg/g and a leukocyte estimate of 11.4%). We observed a statistically significant difference between groups when either 50 μg/g (one tailed t-test, p-value = 4.7E-03) or 250 μg/g (one tailed t-test, p-value = 0.02) were used as the threshold for a positive test; however, there was no significant difference between moderate and high calprotectin samples (one tailed t-test, p-value = 0.07). Our results suggest that human DNA in stool from even a mildly inflamed intestine, as measured by faecal calprotectin, is almost completely derived from leukocytes. In a low inflammatory environment, the proportion of leukocytes in stool is extremely variable, although this might be the result of a relatively small amount of colonic epithelial cells being sloughed out of the lumen.

**FIGURE 4:**
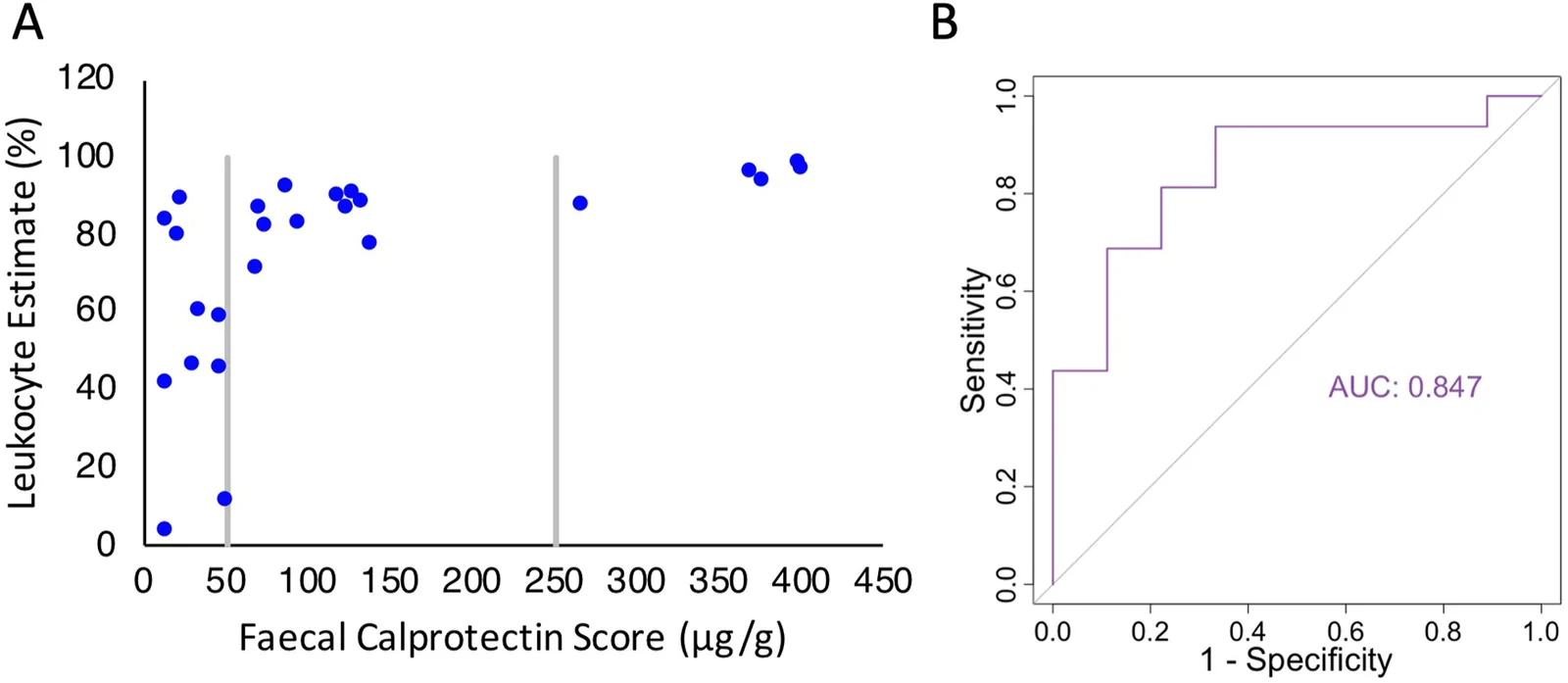
Comparison of the *MAP4K1* bisulfite amplicon sequencing assay to the FCA. **(A)** Scatterplot of leukocyte estimates based on *MAP4K1* methylation in stool samples vs. associated faecal calprotectin score. **(B)** ROC curve for the *MAP4K1* bisulfite amplicon sequencing assay using FCA as the reference standard.

We next examined how well the methylation assay could function as a diagnostic marker. Since we did not have access to the clinical outcomes of each patient, we used the calprotectin level. With a 50 μg/g threshold, the FCA has a sensitivity and specificity of approximately 97% and 80%, respectively (Laserna-Mendieta and Lucendo, 2019; Degraeuwe et al., 2015; Henderson et al., 2014), so while not perfect, offers some indication of assay applicability. We constructed an ROC curve and observed an area under the curve of 0.847, indicating an high level of agreement between assays (Figure 4B). Using these data, the optimal threshold for the methylation assay to identify elevated FCA is a leukocyte estimate of ≥54.9%, with a sensitivity of 0.94 and specificity of 0.67. While promising, future studies will need to apply this in the context of clinical outcomes.

## Discussion

In this proof-of-concept study we have applied two previously identified pan-leukocyte biomarkers (Dunnet et al., 2022), one each at the *HOXA3* and *MAP4K1* loci, to clinically relevant tissue samples: breastmilk and stool. With high-throughput sampling of breastmilk, we have shown that the proportion of leukocytes is extremely variable over short periods of time and between each breast. In the process, we have incorporated a new primer set into the pipeline, highlighting its ease of use and modifiability. Finally, we have demonstrated that even slight intestinal inflammation results in the majority of stool-derived DNA to be of leukocyte origin. Overall, our results show the assay pipeline can accurately and precisely determine the proportion of leukocytes from low levels of DNA.

### Demonstration of cost-effectiveness

Cost-effectiveness is crucial in screening assay design; an assay that is too expensive to run has limited clinical value regardless of its performance metrics. The total reagent cost of performing either the *HOXA3* or *MAP4K1* dual-index PCR is the sum of costs for DNA extraction, bisulfite conversion, PCR amplification, and DNA sequencing per sample (Tables 2, 3). The per-sample cost is dramatically reduced as more individual samples are multiplexed together; thus, the high-throughput nature of the assay enables consumable cost reduction, down to $23 NZD per stool sample or $11 NZD per breastmilk sample.

**TABLE 2:** Total reagent cost of performing the *HOXA3* and *MAP4K1* bisulfite amplicon sequencing assay. Per-sample costs are calculated as the total cost of purchasing the reagent divided by the proportion of the reagent used for a single reaction. Kit prices are at retail price. All costs are in New Zealand Dollars (NZD).

Reagent	Use	Total cost	Number of reactions	Cost per sample
Zymo Research™ Quick-DNA Fecal/Soil Microbe Miniprep Kit	DNA extraction	$604.00	50	$12.08
Zymo EZ-DNA Methylation Direct MagPrep kit (4 × 96 reactions)	Bisulfite conversion	$1,349.00	384	$3.51
PCR reagents	PCR amplification	$540.00	100	$5.40
iSeq100 v2 cartridge and flow cell	DNA Sequencing	$990.00	1	varies

**TABLE 3:** The cost per sample of the *HOXA3* and *MAP4K1* bisulfite amplicon sequencing assay. Breastmilk cell lysis is performed in conjunction with the bisulfite conversion as part of the Zymo EZ-DNA Methylation Direct MagPrep kit. All costs are in New Zealand Dollars (NZD).

Sample type	Number of multiplex sequencing samples	Per sample DNA extraction cost	Per sample bisulfite conversion cost	Per sample PCR amplification cost	Per sample DNA sequencing cost	Total per sample cost
Stool	50	$12.08	$3.51	$5.40	$19.80	$40.79
	100	$12.08	$3.51	$5.40	$9.90	$30.89
	250	$12.08	$3.51	$5.40	$3.96	$24.95
	500	$12.08	$3.51	$5.40	$1.98	$22.97
Breastmilk	50	-	$3.51	$5.40	$19.80	$28.71
	100	-	$3.51	$5.40	$9.90	$18.81
	250	-	$3.51	$5.40	$3.96	$12.87
	500	-	$3.51	$5.40	$1.98	$10.89

### High-throughput assaying of leukocyte fractions in human breastmilk

Leukocytes comprise a relatively small proportion of cells within human breastmilk. For example, in colostrum, leukocytes make up 8%–10% of total cells but are drastically reduced to approximately 1% of cells after 1-week post-partum (Hassiotou et al., 2013a; Trend et al., 2015). However, the fraction of leukocytes increases during illness to the mother or child, with the highest increase attributed to mastitis, where the percentage can exceed 90% (Hassiotou et al., 2013a). Thus, leukocyte proportions are an adequate measure of the overall health of the mother/infant dyad.

We applied the *HOXA3* and *MAP4K1* DNA methylation biomarkers (Dunnet et al., 2022) to milk samples collected from one individual during a lengthy bout of mastitis and were able to track increases and decreases in the leukocyte fraction with considerable consistency between the two biomarker loci. At times during the study period, leukocyte proportions approached normal baseline levels (Figure 2D); however, mostly we recorded high leukocyte production on account of the ongoing infection and injury.

Over the timepoints where breast-of-origin for milk samples could be tracked, we observed clear differences in leukocyte proportions. The primary site of mastitis (the left breast) maintained very high leukocyte levels throughout the peak of infection and in the days immediately following. Interestingly however, milk from the unaffected right breast produced greater than 90% leukocytes at the peak of the infection in the left breast (Figure 2A), but this dropped to 60% in the days immediately following infection.

Previous studies have shown that the levels of leukocytes in breastmilk increase when either the mother or infant is unwell and are dependent on the illness (Hassiotou et al., 2013a). Gastrointestinal infections and vaginal thrush in the mother induce small increases in leukocyte fraction, as do infant-only infections. However, illness in the mother can induce an increase in leukocyte fractions (Hassiotou et al., 2013a). When the mother has mastitis, leukocyte proportions in the unaffected breast have been reported to increase above baseline levels (Hassiotou et al., 2013a). In our time series, the unaffected right breast had a greatly elevated proportion of leukocytes, perhaps indicative of a non-symptomatic infection or injury in that breast.

Leukocyte fractions in breastmilk have been suggested as a diagnostic marker to assess the health of mother/infant dyads (Hassiotou et al., 2013a). However, cellular composition studies employing flow cytometry often only examine milk from one or two timepoints (Riskin et al., 2012; Indumathi et al., 2013). One study on mastitis follows a case/control study design but does not explore the initiation and recovery from mastitis (Riskin et al., 2012). Furthermore, longitudinal studies frequently collect samples weeks apart (Hassiotou et al., 2013a; Trend et al., 2015; Hassiotou et al., 2013b; Nyquist et al., 2022). Given that the cellular components of human breastmilk are dynamic based on the stage of lactation (Trend et al., 2015), degree of breast fullness (Hassiotou et al., 2013b), infant feeding habits (Witkowska-Zimny and Kaminska-El-Hassan, 2017), the health of the mother infant dyad (Hassiotou et al., 2013a), and that the macronutrients within breastmilk are variable throughout the day (Hahn-Holbrook et al., 2019; Paulaviciene et al., 2020), it is not unexpected that the cell proportions of breastmilk change on a near daily basis. Indeed, the breastmilk samples employed in this study were collected almost daily over a 1-month period where continuous collection enabled the tracking of marked leukocyte dynamics within very short timeframes. High-throughput technology such as DNA methylation biomarkers can enable extensive sampling of breastmilk to characterise the cellular dynamics before, during, and after mastitis events with minimal cost and resources. While the process of bisulphite conversion and DNA sequencing is more time-consuming than flow cytometry for individual samples, batch testing of hundreds of samples simultaneously affords considerable efficiency of testing. As such, there is potential for this technology to be applied in a population-wide screen to track breast-related illness in lactating mothers or to further the understanding of the events leading up to mastitis provides more opportunities to prevent severe illness and guide treatment protocol.

### Benchmarking a DNA methylation-based assay for the detection of leukocytes in stool against the faecal calprotectin assay

Stool is a tissue source for identifying intestinal tract pathologies and the clinical benefits of the FCA are well documented. For example, it may be used to aid diagnosis of infection (Sýkora et al., 2010; Shastri et al., 2008), colorectal cancer (von Roon et al., 2007), coeliac disease (Ertekin et al., 2010), or inflammatory bowel disease (IBD) (Laserna-Mendieta and Lucendo, 2019; Ayling and Kok, 2018). While FCAs often do not determine the cause of inflammation, they are extensively used because of their ability to distinguish between IBD and functional bowel disorders, such as irritable bowel syndrome (IBS) (Banerjee et al., 2015).

Inflammatory bowel disease describes a group of chronic inflammatory diseases affecting the gastrointestinal tract. There are two main types of IBD: ulcerative colitis (UC) and Crohn’s disease (CD). Ulcerative colitis explicitly occurs in the colon and manifests as inflammation of the mucosal layer, while CD can present in any area of the gastrointestinal tract and is distinguished by granulomatous inflammation that can penetrate deep into the surrounding tissues (Ayling and Kok, 2018; Matsuoka and Kanai, 2015; Baumgart and Carding, 2007). In contrast, IBS has no identifiable pathophysiological features, and accordingly, no inflammation (Saha, 2014). Despite this, abdominal pain and diarrhoea are common symptoms in both IBD and IBS. Therefore, an endoscopy, occasionally followed by a histological examination of biopsied tissue, is required to diagnose IBD (Bharadwaj et al., 2018). Unfortunately, endoscopies are time-consuming, highly invasive for the patient (with some risk of complications), and require healthcare resources (Fisher et al., 2011). Using faecal calprotectin to distinguish between IBD and IBS minimises unnecessary endoscopies by screening prospective patients and monitoring patient remission to ensure that current treatment regimens are effective (Ayling and Kok, 2018; van Rheenen et al., 2010). Several meta-analyses have reported high sensitivities and excellent negative predictive values for the FCA (reviewed in Laserna-Mendieta and Lucendo (2019)) for diagnosing and monitoring IBD.

### Rational for benchmarking a new intestinal inflammation detection tool

While the FCA has clinical utility when IBD is suspected, several limitations exist. Most commercial assays recommend ≥50 μg/g of calprotectin as the threshold for a positive test (Ayling and Kok, 2018). However, young children and adults over 65 have a higher baseline faecal calprotectin level. An initial meta-analysis suggested that the sensitivity of the FCA was significantly reduced in paediatric populations (van Rheenen et al., 2010). Contrary to this, more recent meta-analyses demonstrate that paediatric sensitivity is high (0.97–0.98) but specificity is reduced in comparison to adults for a 50 μg/g cut-off (Laserna-Mendieta and Lucendo, 2019; Degraeuwe et al., 2015; Henderson et al., 2014). The FCA has a lower sensitivity for adults over 65, resulting in lower diagnostic accuracy in this demographic (Padoan et al., 2018). Several studies have proposed various increases to cut-off thresholds in these groups, but these have not yet been implemented in a clinical setting (Padoan et al., 2018; Joshi et al., 2010; Mindemark and Larsson, 2012; Ezri and Nydegger, 2011). The exact reason elderly individuals have an increase in faecal calprotectin is currently unknown; however, one possible explanation is ‘inflammaging’ – an age-associated low-grade chronic inflammation in the absence of infection (Leonardi et al., 2018). In our study, methylation-based analysis detected a leukocyte signal in faecal samples that had a negative FCA result. Our previous work (Dunnet et al., 2022) showed specificity of the methylation pattern to leukocytes, whereas calprotectin is relatively specific to neutrophils raising the possibilities that we have detected non-neutrophlic leukocytes, such as monocyte/macrophages, eosinophils or lymphocytes. Alternatively the negative FCA result might reflect some of the factors that result in its low sensitivity.

The stability of faecal calprotectin over time has been extensively studied since the test was first proposed. An initial study reported that faecal calprotectin is stable at room temperature for up to 7 days (Røseth et al., 1992). Since then, several studies have shown that calprotectin is significantly degraded after 48–72 h at 20°C (Haisma et al., 2019; Lasson et al., 2015; Oyaert et al., 2017). Storage at 4°C significantly reduces sample degradation for up to a week (Haisma et al., 2019). Calprotectin stability may prove problematic when samples must be transported long distances to testing facilities (for example, from rural communities) unless samples can be chilled during travel. In contrast, DNA methylation is a very stable epigenetic mark, although DNA itself is susceptible to hydrolysis when stored in an aqueous solution. However, storage of DNA in an appropriate conservation buffer can drastically decrease degradation. For example, in an experiment measuring DNA stability in stool samples, Olson et al. (2005) (Olson et al., 2005) show no DNA loss after 144 h of storage (the longest time point measured) at room temperature in three of four buffers tested. Their data suggest that DNA can be stable at least twice as long as calprotectin at room temperature.

Measuring faecal calprotectin is performed with an enzyme immunoassay, and each manufacturer uses unique proprietary antibodies. Different FCAs correlate well to patient outcomes, but absolute values cannot be compared between assays (Laserna-Mendieta and Lucendo, 2019; Ayling and Kok, 2018). For example, one study showed a 3.8-fold difference in the amount of calprotectin measured by different assays in a quality assurance scheme sample (Whitehead et al., 2013), while significant inter-assay differences have been observed in paediatric and adult IBD cases (Kittanakom et al., 2017; Labaere et al., 2014). Therefore, to achieve accurate follow-up testing during patient treatment, medical testing facilities must use a standardised FCA (Ayling and Kok, 2018). Alternatively, this may be alleviated with an open-source PCR-based DNA methylation assay where primer sequences and reaction conditions are known.

Calprotectin is also elevated in patients with pancreatic insufficiency (such as those with cystic fibrosis) (Ellemunter et al., 2017). This is likely due to a reduction of trypsin, which readily degrades calprotectin (Dumoulin et al., 2015). Here, a DNA methylation-based approach to identify leukocyte DNA might provide a more accurate representation than faecal calprotectin.

In healthy individuals, host DNA in stool is thought to predominately originate from sloughed colonic epithelial cells (He et al., 2019); however, cancers of the intestinal tract also shed DNA into the intestinal lumen (Whitney et al., 2004). Amplification of stool-derived DNA has been proposed as a non-invasive cancer detection tool (Mojtabanezhad Shariatpanahi et al., 2018). We hypothesized that the *MAP4K1* pan-leukocyte DNA biomarker coupled with the our high-throughput pipeline would detect an increase in leukocytes during inflammation which may be clinically relevant. Indeed, the high level of agreement between our pipeline and the FCA emphasises its clinical potential. We have shown that under even mild inflammatory states (as assessed by FCA), DNA from leukocytes is the dominant fraction of human-derived DNA. Interestingly, low calprotectin stool samples (under 50 μg/g) exhibited extremely variable proportions of leukocytes. This observation could be the result of a relatively low amount of sloughed epithelial cells such that even minor increases in leukocytes dramatically alters the overall proportion of cell types in each sample. Alternatively, this could be attributed to imprecisions in either the faecal calprotectin or DNA methylation assays. Patients with high levels of intestinal inflammation contain increased human DNA in their stool (Vincent et al., 2015), so it is likely the assay will perform better under higher levels of inflammation where more DNA is accessible for amplification. The FCA has high negative predictive value, but has been reported to generate a significant number of false positives (Freeman et al., 2021). As such, one possible application of our pipeline is for it to be used in tandem with the FCA to reduce the number of false positive results and subsequently the number of patients requiring an endoscopy.

## Conclusion

In conclusion, we have applied a high-throughput, cost-effective DNA methylation biomarker pipeline to identify leukocytes from a mixed tissue. By employing a rapid sampling approach, we show that leukocyte proportions in breastmilk vary greatly with short periods of time. Leukocyte proportions in breastmilk are a useful health indictor of the mother/infant dyad, so a rapid sampling approach may provide the most accurate clinical information in this context. In addition, we have also shown that leukocyte-derived DNA can be detected in stool using our pipeline. We observed that even with low levels of intestinal inflammation as measured by faecal calprotectin, leukocyte-derived DNA dominates the human DNA fraction in stool samples.

## Data Availability

The datasets presented in this study can be found in online repositories. The names of the repository/repositories and accession number(s) can be found below: https://github.com/TimHore-Otago/Dunnet_NZ_Pan-leukocyte_Inflammation_Biomarkers.

## Abbreviations

CpG, CG dinucleotide; cfDNA, cell-free DNA; FCA, Faecal Calprotectin Assay; ROC, Receiver Operating Characteristic; AUC, Area Under the Curve; IBD, Inflammatory Bowel Disease; IBS, Irritable Bowel Syndrome; UC, Ulcerative Colitis; CD, Crohn’s Disease; PEG, polyethylene glycol.
